# Praxis der präklinischen Schlaganfallversorgung im deutschsprachigen Raum: Eine Umfrage unter den ärztlichen Leitungen Rettungsdienst in Deutschland, Österreich und der Schweiz

**DOI:** 10.1007/s10049-022-01112-x

**Published:** 2023-01-20

**Authors:** Martin Lier, Maximilian Euler, Markus Roessler, Jan Liman, Meike Bettina Goericke, Michael Baubin, Stefan Matthias Mueller, Nils Kunze-Szikszay

**Affiliations:** 1 Klinik für Anästhesiologie, Universitätsmedizin Göttingen, Robert-Koch-Straße 40, 37075 Göttingen, Deutschland; 2 Klinik für Neurologie, Universitätsmedizin Göttingen, Robert-Koch-Straße 40, 37075 Göttingen, Deutschland; 3 Klinik für Neurologie, Klinikum Nürnberg, Universitätsklinik der Paracelsus Medizinischen Privatuniversität, Breslauer Straße 201, Nürnberg, 90471 Deutschland; 4 Universitätsklinik für Anästhesie und Intensivmedizin, Medizinische Universität Innsbruck, Anichstraße 35, 6020 Innsbruck, Österreich; 5 Schutz & Rettung, Stadt Zürich, Neumühlequai 40, 8021 Zürich, Schweiz

**Keywords:** Rettungsdienst, Schlaganfall, Thrombektomie, Versorgungsleitlinie, Diagnosescores, Emergency medical services, Stroke, Thrombectomy, Clinical guidelines, Diagnostic scores

## Abstract

**Hintergrund:**

Eine leitlinienadhärente rettungsdienstliche Versorgung kann die Prognose von Schlaganfallpatienten positiv beeinflussen.

**Ziel der Arbeit:**

Durchführung einer Bestandsaufnahme der Organisation der präklinischen Schlaganfallversorgung im Hinblick auf die Empfehlungen aktueller Versorgungsleitlinien.

**Material und Methoden:**

Die ärztlichen Leitungen Rettungsdienst (ÄLRD) in Deutschland (*n* = 178), Österreich (*n* = 9) und der Schweiz (*n* = 32) wurden zu einer Onlinebefragung (unipark.com, Tivian XI GmbH, Köln, Deutschland) eingeladen. Die Umfrage war über 10 Wochen (22.04. bis 30.06.2020) erreichbar, erfolgte anonym und schloss Angaben zu Strukturdaten, zur klinischen Versorgung und zur Alarmierungs- bzw. Versorgungsstrategie ein.

**Ergebnisse:**

Die Umfrage wurde 69-mal beendet und 65 Datensätze in die Auswertung einbezogen (4-mal kein ÄLRD). Die Merheit von 73,8 % (*n* = 48) waren ÄLRD in Deutschland, 15,4 % (*n* = 10) in der Schweiz und 10,8 % (*n* = 7) in Österreich. Es ergaben sich wesentliche Unterschiede in der infrastrukturellen Ausstattung der RD-Bereiche: 93,3 % (*n* = 61) der Befragten gaben an, eine SOP zur allgemeinen Schlaganfallversorgung zu nutzen, 37 % (*n* = 24) unterschieden zwischen Schlaganfällen mit leichter und schwerer Symptomatik und 15,4 % (*n* = 10) nutzten einen spezifischen Score zur Vorhersage von proximalen Gefäßverschlüssen mit hoher Thrombektomiewahrscheinlichkeit.

**Diskussion:**

Die präklinische Schlaganfallversorgung ist sehr heterogen organisiert. In Hinblick auf Leitlinienadhärenz und die Einschätzung der Thrombektomiewahrscheinlichkeit besteht, unter anderem durch einheitliche Nutzung präklinischer Vorhersagescores, ein erhebliches Optimierungspotenzial.

**Graphic abstract:**

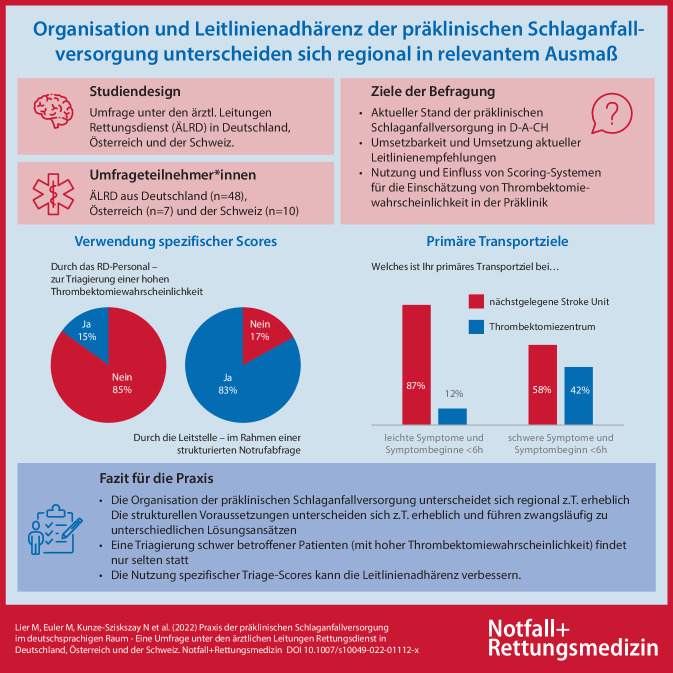

## Abstract (en)

**Background:**

The prognosis of stroke patients can be improved by adherence to clinical guidelines.

**Objective:**

To analyse the current state of organisation of prehospital stroke treatment in Germany, Austria and Switzerland with a focus on guideline adherence.

**Materials and methods:**

All medical directors of emergency medical services (MDEMS) in Germany (*n* = 178), Austria (*n* = 9) and Switzerland (*n* = 32) were invited to complete an anonymous online survey (unipark.com, Tivian XI GmbH, Cologne, Germany) which was available for 10 weeks from April–June 2020. Participants were asked for information regarding structural organisation, clinical treatment and strategic/tactical aspects.

**Results:**

The survey was completed 69 times and 65 datasets were analysed (4 participants without MDEMS status): 73.8% (*n* = 48) were MDEMS from Germany, 15.4% (*n* = 10) from Switzerland and 10.8% from Austria (*n* = 7). The survey results show relevant differences in the infrastructure of and the approach to prehospital stroke treatment. Standard operating procedures for stroke treatment were in place in 93.3% (*n* = 61) of the EMS areas. Furthermore, 37% (*n* = 24) of the EMS areas differentiated between stroke with mild and severe symptoms and 15.4% (*n* = 10) used specific scores for the prehospital prediction of large vessel occlusion strokes (LVOS).

**Conclusions:**

Our data highlight the heterogeneity of prehospital stroke treatment in Germany, Austria and Switzerland. Consistent use of appropriate scores for LVOS prediction and a higher adherence to recent clinical guideline in general are measures that should be taken to optimise the prehospital treatment of stroke patients.

## Kurze Hinführung zum Thema

Der Schlaganfall ist eine der häufigsten Ursachen für Mortalität und Behinderung weltweit [7]. Eine aktuelle Studie gibt das Lebenszeitrisiko für Mitteleuropäer, einen Schlaganfall zu erleiden, mit 31,6 % an [6]. Allein in Deutschland treten jährlich etwa 270.000 Krankheitsfälle auf [28]. Der präklinischen Versorgung von Schlaganfallpatienten kommt schon aufgrund des häufigen Auftretens eine besondere Bedeutung zu. Schnelle adäquate Diagnostik und Therapiebahnung durch den Rettungsdienst kann wesentlich zum Behandlungserfolg beitragen [39].

## Hintergrund und Fragestellung

Die herausragende Evidenzlage für die mechanische Rekanalisationstherapie mittels Thrombektomie, insbesondere bei proximalen Gefäßverschlüssen, führte um das Jahr 2015 zu einem Paradigmenwechsel in der Schlaganfalltherapie [11]. In der Folge kam es rasch zur Anpassung nationaler und internationaler Therapieleitlinien [24, 29]. Der ischämische Schlaganfall bleibt ein zeitkritisches Krankheitsbild, wobei insbesondere das Zeitintervall von 6 h nach Symptombeginn die kritische Zeitgrenze für eine direkte endovaskuläre Thrombektomie darstellt [24, 28]. Ziel dieser Befragung war es, eine Momentaufnahme der präklinischen Schlaganfallversorgung im deutschsprachigen Raum zu erhalten. Hierbei sollten neben dem präklinischen Vorgehen vor allem auch spezifische Zuweisungsstrategien abgefragt werden. Die Ergebnisse der Umfrage wurden mit den Empfehlungen aktueller nationaler und internationaler Versorgungsleitlinien abgeglichen und in deren Kontext gesetzt.

## Studiendesign und Untersuchungsmethoden

Die ärztlichen Leitungen Rettungsdienst (ÄLRD) in Deutschland, Österreich und der Schweiz wurden zur Teilnahme an einer Onlineumfrage eingeladen [35]. Die Kontaktaufnahme erfolgte in Deutschland über die dienstlichen Mailadressen aller uns bekannten ÄLRD (*n* = 178; zugänglich u. a. über die Webseite des Bundesverbands der ÄLRD www.bv-aelrd.de), in Österreich ebenfalls direkt an alle zuständigen Ärzt*innen in den Bundesländern (*n* = 9) und in der Schweiz über die Schweizerische Gesellschaft für Notfall- und Rettungsmedizin (*n* = 32). Die Onlineumfrage war über 10 Wochen im Zeitraum vom 22.04. bis zum 30.06.2020 erreichbar. Die Teilnahme an der Umfrage erfolgte anonym. Der Rettungsdienstbereich konnte genannt werden (freiwillige Angabe). Zielgruppe der Umfrage waren ausschließlich ärztliche Kolleg*innen, die für einen Rettungsdienstbereich organisatorisch verantwortlich waren. Zu Beginn der Umfrage wurde diese Organisationsverantwortung mit einem Pflichtfeld abgefragt.

Folgende Parameter wurden im Rahmen der Onlineumfrage erfasst:

- Strukturdaten zum Rettungsdienstbereich (u. a. Land, Einwohnerzahl, Art und Anzahl der Rettungsmittel, universitäre Anbindung, Einsatzzahlen für 2019);
- Daten zur klinischen Versorgungsstruktur (u. a. Anzahl, Art und Verfügbarkeit von Stroke Units und neuroradiologischen Interventionsmöglichkeiten);
- Alarmierungs- und Versorgungsstrategie für die Schlaganfallversorgung (u. a. strukturierte Notrufabfrage, primäre Notarzteinsatzindikation, Vorhandensein von Standard Operating Procedures [SOP] zur Schlaganfallversorgung mit Bezug auf leitlinienbezogene Versorgungskategorien, Zuweisungsstrategien mit Bezug auf leitlinienbezogene Versorgungskategorien).

Die Auswertung der Daten und graphische Darstellung erfolgte per SPSS Statistic Version 27 (IBM, Armonk, NY, USA) die graphische Darstellung per Microsoft Excel Professional Plus 2019 (Microsoft, Redmond, WA, USA).

## Ergebnisse

Im Erfassungszeitraum wurde die Umfrage insgesamt 173-mal aufgerufen und 69-mal (39,8 %) beendet. Vier Datensätze wurden ausgeschlossen, da die Frage nach der Organisationsverantwortung für den Rettungsdienstbereich verneint wurde. Die übrigen 65 Datensätze wurden in die Auswertung einbezogen.

### Strukturdaten zum Rettungsdienstbereich

**Figure 1:**
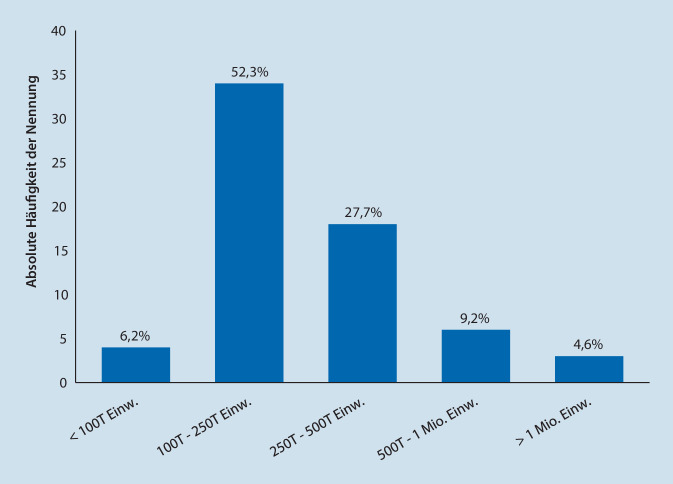

Der Großteil der Befragten stammte aus Deutschland (*n* = 48; 73,8 %), gefolgt von der Schweiz (*n* = 10; 15,4 %) und Österreich (*n* = 7; 10,8 %). Die Einwohnerstruktur der Versorgungsgebiete umfasste Gebiete aller vorgegeben Größenstufen (< 100.000 bis > 1 Mio. Einwohner). 80 % der Befragten gaben an, für ein Gebiet mit einer Einwohnerzahl von 100.000–500.000 Einwohnern zuständig zu sein (siehe hierzu Abb. 1).

In 23,1 % der Gebiete war wenigstens ein Notarztstandort an eine Universitätsklinik angebunden. Zum Zeitpunkt der Erhebung verfügte jede 10. Region über ein Telenotarztsystem. Zwei ÄLRD (3,1 %) gaben an, über eine mobile Stroke Unit (Rettungsmittel mit mobiler Computertomographie und Lysemöglichkeit) zu verfügen.

### Daten zur klinischen Versorgungsstruktur

**Figure 2:**
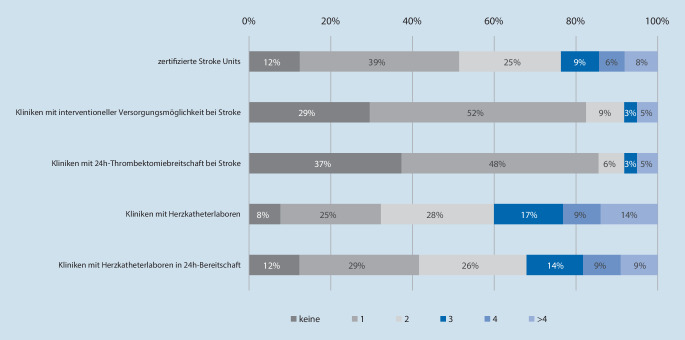

Abb. 2 gibt einen Überblick über die Versorgungsstrukturen im klinischen Sektor zum Zeitpunkt der Befragung. Ein geringer Anteil der Versorgungsgebiete verfügte zum Zeitpunkt der Befragung nicht über eine Stroke Unit (*n* = 5; 7,7 %) bzw. über keine zertifizierte Stroke Unit (*n* = 8; 12,5 %). In etwa einem Drittel der befragten Rettungsdienstbereiche stand kein Thrombektomiezentrum zur Verfügung (*n* = 19; 29 %) oder das vorhandene Thrombektomiezentrum verfügte nicht über eine 24 h-Bereitschaft (*n* = 24, 37 %).

### Alarmierungs- und Versorgungsstrategien für die Schlaganfallversorgung

Mehrheitlich erfolgte eine standardisierte Notrufabfrage durch die Leitstellendisponenten (*n* = 37; 57 %). In Regionen mit standardisierter Notrufabfrage wurde sehr oft (83 %) ein spezifischer Schlaganfallscore (zumeist Face-arm-speech-Test [FAST]) zur standardisierten Abfrage spezifischer Symptome genutzt.

25 ÄLRD (38,5 %) gaben an, dass die Notfallmeldung „Verdacht auf Schlaganfall/Apoplex“ eine primäre Notarzteinsatzindikation ist, sodass die Rettungsleitstellen primär ein arztbesetztes Rettungsmittel (Notarzteinsatzfahrzeug, Rettungshubschrauber) zur Einsatzstelle disponieren. Entsprechend ergab sich ein Gefälle bei der Notarztbeteiligung. Diese betrug über alle Bereiche hinweg 49 % und schwankte zwischen 28 % in Bereichen ohne primäre Notarztalarmierung bis zu 71 % in Bereichen mit regelhafter Notarztalarmierung.

**Figure 3:**
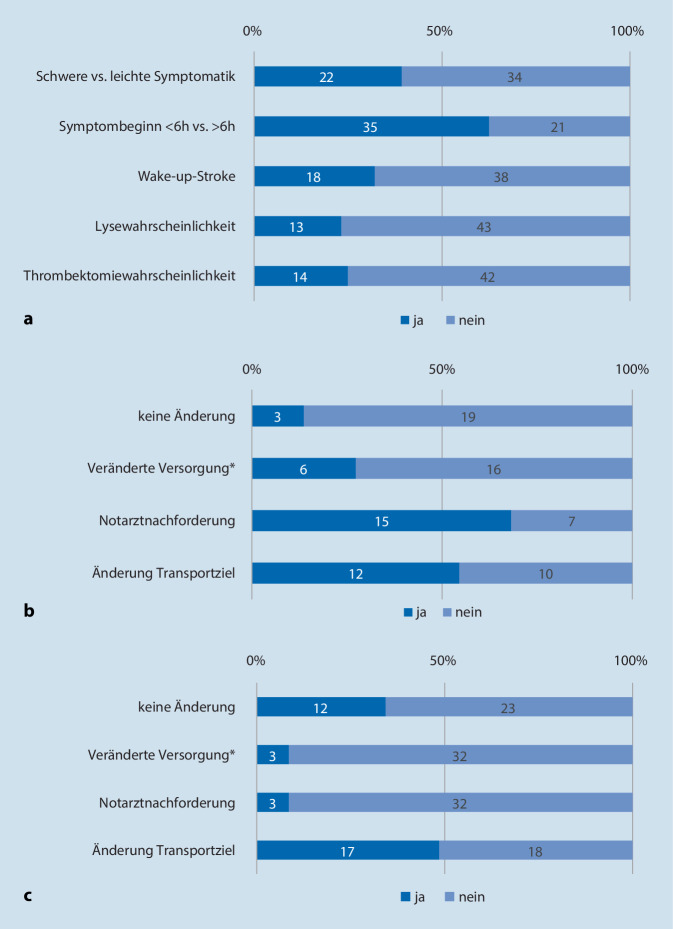

Fast alle Bereiche (93 %) verfügten über eine Standardvorgehensweise (SOP) zur präklinischen Schlaganfallversorgung. Es zeigte sich, dass vor allem die Schwere der Symptomatik (leicht oder schwer betroffen) und das Zeitfenster seit Symptombeginn (bis 6 h oder darüber hinaus) eine veränderte Versorgung durch den Rettungsdienst (Notarztnachforderung, verändertes Transportziel) triggerten (siehe Abb. 3).

15,4 % der Befragten (*n* = 10) gaben an, für *schwere* Schlaganfälle eine *spezifische* SOP zu nutzen. Das Einführen dieses zusätzlichen Behandlungspfads wurde vor allem mit veränderten Leitlinienempfehlungen begründet. Auch in Bereichen, die zum Zeitpunkt der Befragung eine solche spezifische SOP eingeführt hatten, erfolgt in nur etwa der Hälfte der Bereiche ein direkter Transport schwer betroffener Schlaganfallpatienten zu einer Klinik mit Thrombektomieoption. Begründet wurde dies mit langen Transportwegen oder der Nutzung etablierter Konzepte zur initialen Versorgung (Bildgebung, ggf. Lysetherapie) mit sekundärer Verlegung in ein Thrombektomiezentrum (sog. Drip-and-ship-Konzepte). Rettungsdienstbereiche mit spezifischer SOP für schwere Schlaganfälle verfügten über eine vergleichbare Anzahl (zertifizierter) Stroke Units und Thrombektomiezentren im Vergleich zu Bereichen ohne entsprechende SOP. Insbesondere fanden sich auch unter den Rettungsdienstbereichen mit eigener SOP für schwere Schlaganfälle Gebiete, in denen keine Thrombektomiezentren existieren (20 % der Regionen mit vs. 25 % der Regionen ohne eigene SOP für schwere Schlaganfälle).

Unabhängig von Angaben zu Standardvorgehensweisen wurden die Teilnehmenden konkret zu Transportzielen leitlinienrelevanter Fallkonstellationen befragt. Hierbei zeigte sich, dass vor allem die Schwere der Symptomatik Einfluss auf die Auswahl des Transportziels hat. Betroffene mit leichten Symptomen (z. B. milde, isolierte Kraftminderung eines Arms und/oder Dysarthrie) würden demnach unabhängig vom Symptombeginn in 80–87 % der Fälle zur nächstgelegenen Stroke Unit transportiert. Im Gegensatz dazu machten die Befragten bei einer schweren Symptomatik (z. B. deutliche Hemiparese und/oder Aphasie und/oder Neglect) das Transportziel in relevantem Umfang vom Symptombeginn abhängig. Betroffene mit einem Symptombeginn vor 6 h oder weniger würden demnach in 42 % der Fälle direkt dem nächstgelegenem Thrombektomiezentrum zugeführt. Bei einer Symptomdauer von 6–24 h gaben dies noch 33 % der Befragten an. Bei einem Schlaganfall mit unbekannter Symptomdauer (z. B. *Wake-up Stroke*) würden ca. 40 % der Fälle direkt zum Thrombektomiezentrum transportiert.

**Figure 4:**
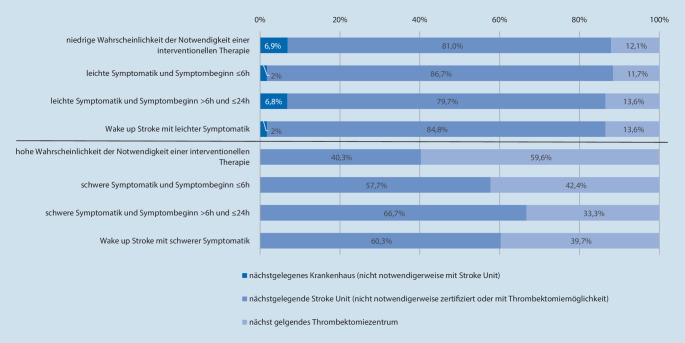

Aktuelle Versorgungsleitlinien empfehlen eine Akutthrombektomie mit hohem Evidenzgrad und ohne vorherige Spezialbildgebung (z. B. Perfusions-CT) für Verschlüsse proximaler hirnversorgender Blutgefäße der vorderen Strombahn (Arteria carotis, proximale Arteria cerebri media) mit einer Symptomdauer bis zu 6 h [25]. Diese Konstellation wurde mit 2 verschiedenen Formulierungen abgefragt, wobei fast 60 % der Befragten angaben, Betroffene „mit einer hohen Wahrscheinlichkeit für die Notwendigkeit einer interventionellen Therapie“ direkt in ein Thrombektomiezentrum zu transportieren. Bei einer weniger impliziten Formulierung der gleichen Konstellation, nämlich „schwere Symptomatik (z. B. deutliche Hemiparese und/oder Aphasie und/oder Neglect) mit Symptombeginn ≤ 6 h“ gaben dies dagegen nur 42 % an (siehe Abb. 4).

## Diskussion

Mit dem Aufkommen der mechanischen Revaskularisierung erfuhr die Therapie des akuten ischämischen Schlaganfalls seit 2015 einen Paradigmenwechsel [3, 11]. Der Stellenwert der systemischen Thrombolysetherapie bleibt zwar bestehen, die Evidenzlage für die prognostische Überlegenheit der Thrombektomie, wenn möglich in Kombination mit einer Lysetherapie, ist jedoch überragend [27]. Als Ende 2019 die vorliegende Umfrage konzipiert wurde, empfahl die Leitlinie der American Heart Association/American Stroke Association den Einsatz der mechanischen Revaskularisierung bei arteriellen Verschlüssen der vorderen Strombahn (Arteria carotis, Arteria cerebri media) bei einer Symptomdauer bis 6 h und darüber hinaus [25]. Die Möglichkeit zur endovaskulären Therapie stellt die Organisation der präklinischen Versorgung von Schlaganfallpatienten vor neue Herausforderungen. Vom Erstkontakt bei der Notrufabfrage über die eigentliche medizinische Versorgung bis hin zur korrekten Auswahl der Zielklinik – der rettungsdienstlichen Organisation fällt eine entscheidende Bedeutung für den Therapieerfolg der Betroffenen zu. Die erheblich veränderten Abläufe im klinischen Versorgungssektor müssen notwendigerweise Veränderungen in den Prozessen des präklinischen Sektors nach sich ziehen.

Die vorliegende Umfrage stellt eine Bestandsaufnahme dieser Prozesse zu Beginn der SARS-CoV-2-Pandemie im Frühjahr 2020 dar. Die ÄLRD sind verantwortlich für die Prozessorganisation der Rettungsdienstbereiche und waren daher Zielgruppe dieser Umfrage. Wir erachten die beschriebene Kohorte als relevant groß und ausreichend repräsentativ, da die Verteilung der Einwohnerzahlen der teilnehmenden Rettungsdienstbereiche nahezu der Bevölkerungsverteilung der Landkreise und kreisfreien Städte der Bundesrepublik Deutschland entspricht [33]. Inwieweit die Teilnehmenden eine repräsentative Verteilung bezogen auf Bundesländer oder Kantone darstellen, lässt sich aufgrund der Anonymisierung der Befragung nicht sicher sagen. Weiterhin muss eingeschränkt werden, dass zwar eine Empfehlung der Bundesärztekammer zur Funktion und Kompetenz des ärztlichen Leiters Rettungsdienst existiert, inwieweit diese tatsächlich entsprechend umgesetzt wird, ist nicht zuletzt eine Frage der örtlichen Rettungsdienstgesetze. Hieraus ergeben sich in der Folge auch Einschränkungen in der Weisungsbefugnis gegenüber beispielsweise in der jeweiligen Körperschaft tätigen Notärzte.

Im vergangenen Jahr erfolgte mit der Veröffentlichung der S2e-Leitlinie „Akuttherapie des ischämischen Schlaganfalls“ auch die Aktualisierung der deutschsprachigen Versorgungsleitlinien, mit der auch konkrete Empfehlungen für die präklinische Versorgung formuliert wurden [29]. Die vorliegenden Daten erlauben eine Einordnung des Organisationszustands der Rettungsdienste im deutschsprachigen Raum in Bezug auf die Leitlinienempfehlungen. Tab. 1 fasst die für die präklinische Notfallmedizin relevanten Empfehlungen der Leitlinie zusammen.

**Table 1:** 

LoE	Empfehlung
2	Bei Patienten mit akut aufgetretenen neurologischen Symptomen sollte eine etablierte Skala, beispielsweise Face-arm-speech-Test (FAST) angewendet werden, um prähospital nach einem Schlaganfall zu screenen
3	Die Verwendung einer validierten Skala zur Bestimmung der Schlaganfallschwere kann empfohlen werden, da dies Einfluss auf Transportziel und -modalität haben kann
Statement: Der Verschluss eines großen intrakraniellen Gefäßes ist auch bei Vorliegen der Kombination von Hemiparese und kortikalen Symptomen (z. B. Aphasie, Neglect, Blickparese) wahrscheinlich	
EK	Derzeit kann keine Empfehlung ausgesprochen werden, mit welcher klinischen Skala Schlaganfallpatienten identifiziert werden können, die einen endovaskulär behandelbaren Gefäßverschluss aufweisen
EK	Da es keine eindeutigen Beweise für die Überlegenheit eines der Organisationsmodelle (z. B. „drip and ship“, „mothership“, „drip and drive“) gibt, sollte die Wahl des Modells von der lokalen und regionalen Dienstleistungsorganisation und den Patientencharakteristika abhängen
EK	Das Mothership-Modell könnte in Ballungsgebieten mit einer Transportzeit zu einem Comprehensive Stroke Center von unter 30–45 min bevorzugt werden; das Drip-and-ship-Systems hingegen, wenn die Transportzeit länger ist
1	Bei Patienten mit akutem ischämischem Schlaganfall, klinisch relevantem neurologischem Defizit und Verschluss einer großen Arterie im vorderen Kreislauf soll, wenn innerhalb von 6 h (Zeit zwischen Symptombeginn und Leistenpunktion) möglich, eine mechanische Thrombektomie erfolgen, um das funktionelle Ergebnis zu verbessern
Statement: Die beste Evidenz für die endovaskuläre Schlaganfalltherapie besteht für Patienten mit prä-mRS 0–1, ursächlichem Verschluss der A. carotis interna und oder des Mediahauptstamms (M1-Segment), Alter ≥ 18 Jahre, NIHSS ≥ 6 und ASPECTS ≥ 6. Das bedeutet aber nicht, dass die EST nicht auch bei Patienten effektiv sein kann, auf die diese Kriterien nicht zutreffen	
2	Eine mechanische Thrombektomie sollte auch erfolgen, wenn ein oder mehrere M2-Segmente betroffen sind
EK	Eine mechanische Thrombektomie kann auch bei Verschlüssen der A. cerebri anterior oder der A. cerebri posterior von Vorteil sein
1	Jenseits des 6 h-Zeitfensters soll eine mechanische Thrombektomie relevanter Verschlüsse im vorderen Kreislauf erfolgen, wenn durch erweiterte Bildgebung (z. B. Darstellung eines kleinen Infarktkerns, Mismatch, Kollateraldarstellung) im Kontext der klinischen Symptomatik zu vermuten ist, dass rettbares Risikogewebe vorliegt
1	Um einen möglichst großen Nutzen zu erzielen, soll eine Reperfusion so früh wie möglich innerhalb des therapeutischen Zeitfensters erreicht werden

Level of Evidence (*LoE*): 1–5 nach Oxford Centre of Evidence Based Medicine [22]

*LoE* Level of Evidence, *EST* endovaskuläre Schlaganfalltherapie

Internationale Leitlinien empfehlen die Nutzung etablierter Skalen zum Schlaganfallscreening bereits bei der telefonischen Abfrage des Notrufs [25]. Obwohl dies in der deutschsprachigen Leitlinie keine Erwähnung findet, gaben fast 57 % der ÄLRD für ihren Bereich an, derartige Skalen im Rahmen einer standardisierten Notrufabfrage zu nutzen. Standardisierte Abfragen können die Effizienz des frühesten Glieds der Rettungskette wesentlich erhöhen und damit zum Behandlungserfolg beitragen. Eine Arbeit aus den USA konnte zeigen, dass 40 % der Schlaganfallpatienten ihr Krankheitsbild selbst fehldeuten [18]. Die Abfrage schlaganfallspezifischer Symptome durch Leitstellendisponenten basiert meist auf FAST und kann den Anteil korrekter Alarmierungsstichworte damit relevant erhöhen [2, 5]. Für Österreich konnte bereits 2015 gezeigt werden, dass eine konsequente Abfrage schlaganfallspezifischer Symptome zur korrekten Klinikzuweisung der Betroffenen beitragen kann [37].

Mit 93 % gaben fast alle Teilnehmenden an, eine Standardvorgehensweise zur präklinischen Schlaganfallversorgung zu nutzen. Der Großteil der angegebenen SOP berücksichtigte den Symptombeginn und die Symptomschwere als Kriterien für eine Anpassung der klinischen Versorgung. Auffällig war, dass alle Teilnehmenden ein Zeitfenster von 6 h seit Symptombeginn als Entscheidungsgrundlage für eine veränderte Versorgung (meist verändertes Transportziel, vgl. Abb. 3) angaben. Die Implementierung dieser Kriterien ist wichtige Voraussetzung für die Adhärenz zu den Leitlinien.

Die Leitlinienempfehlungen spiegeln die Komplexität der Anforderungen wider, denen sich der präklinische Sektor ausgesetzt sieht. Denn Ziel des Rettungsdiensts darf nicht nur das Erkennen allgemeiner Schlaganfallsymptome sein. Vielmehr wird die Forderung erhoben, dass bereits präklinisch die Einordnung der Betroffenen in die korrekte Behandlungskategorie erfolgen soll. Die vorliegenden Daten zeigen, dass die Einschätzung der Thrombektomiewahrscheinlichkeit bisher nur selten erfolgt und die Nutzung spezifischer Scoring-Systeme zur Vorhersage von proximalen Gefäßverschlüssen wenig verbreitet ist. Gute Sensitivitäts- und Spezifitätswerte weisen hierbei vor allem Scoring-Systeme auf, die bereits gut etablierte Items aus dem FAST mit kortikalen Items, wie die Beurteilung einer Herdblick- oder Neglectsymptomatik, kombinieren. Beispiele für etablierte Scores sind der Cincinatti Prehospital Stroke Scale (CPSS), der Gaze-face-arm-speech-Test (G-FAST) oder der Field Assessment Stroke Triage for Emergency Destination (FAST-ED Score; [14, 17, 31]). Durch Nutzung dieser Skalen, die alle auf Items der sehr komplexen NIHSS basieren, kann ein Großteil proximaler Gefäßverschlüsse bereits präklinisch identifiziert werden [38]. In einer aktuellen Arbeit aus Helsinki schnitt der FAST-ED Score vor allem in Hinblick auf positive und negative prädiktive Werte sehr gut ab [17, 26]. Die in Österreich entwickelte und in mehreren Bundesländern genutzte Austrian Prehospital Stroke Scale (APSS) schließt bei vorhandener Armlähmung auch die Beurteilung des seitengleichen Beins sowie das kortikale Item Herdblick ein und zeigte sich in einer aktuellen Arbeit den vorgenannten Scores sogar überlegen [15].

Die Angaben der ÄLRD in der Umfrage ergaben ein heterogenes Bild beim Management der durch die Leitlinien definierten Versorgungskategorien. Demnach werden über 90 % der Patienten mit einer Schlaganfallsymptomatik in eine Stroke Unit oder ein Thrombektomiezentrum transportiert. Insbesondere bei Schlaganfällen im kritischen 6 h-Zeitfenster oder mit unklarem Symptombeginn wurde dies sogar in mehr als 98 % der Fälle angegeben. Im Eckpunktepapier für die notfallmedizinische Versorgung der deutschen Bevölkerung wurde 2016 der Schlaganfall weiterhin als sog. Tracerdiagnose klassifiziert und die Vorgaben für die Versorgung festgelegt [8]. Ebenfalls wurde nochmals die Notwendigkeit einer Zuführung von Patienten mit einer akuten Schlaganfallsymptomatik in die nächstgelegene, zertifizierte Stroke Unit unterstrichen. Die aktuelle Umfrage zeigt hingegen, dass bis zu einem Drittel der Patienten mit leichter Symptomatik und bis zu 20 % der schwer betroffenen Patienten in die nächstgelegene Klinik und nicht zwingend in eine zertifizierte Stroke Unit transportiert werden. Das wird unter anderem dadurch erklärt, dass 12 % der Befragten in ihrem Rettungsdienstbereich nicht über eine zertifizierte Stroke Unit verfügen. Dieses Ergebnis deckt sich mit einer Untersuchung des Bundesinstitutes für Bau, Stadt- und Raumforschung (BBSR) aus dem Jahr 2020, in der 11 % der deutschen Bevölkerung keine zertifizierte Einrichtung binnen 30-minütiger Fahrzeit erreichen können. Jedoch wohnen nur 0,5 % der Bevölkerung mehr als 60 min Fahrtzeit von der nächsten zertifizierten Stroke Unit entfernt [16]. Vor allem für diese Rettungsdienstbereiche ist der Aufbau eines effizienten Versorgungsnetzwerks unter Beteiligung präklinischer und klinischer Akteure zu fordern. Telemedizinische Schlaganfallnetzwerke leisten einen wesentlichen Beitrag in der flächendeckenden Schlaganfallversorgung in nicht urbanen Regionen [1]. Womöglich bestehen solche Infrastrukturen in den Bereichen, die Patienten mit Verdacht auf Schlaganfall nicht primär einer Stroke Unit zuführen. Mit regional adaptierten Verlegungskonzepten nach dem Drip-and-ship- bzw. Mothership-Prinzip, die noch weiter wissenschaftlich beleuchtet werden müssen, kann der Zeitverlust durch Sekundärverlegungen minimiert werden [9, 19, 23]. Die Rettungsdienste müssen hierbei als medizinisch-logistisches Bindeglied zwischen den Behandlungsstrukturen unbedingt eingebunden sein.

Die AHA/ASA-Leitlinie fordert die Einrichtung von regionalen, intersektoralen Strukturen für die Versorgung von Schlaganfällen unter Einbeziehung sowohl von Kliniken mit der Möglichkeit zur Lysetherapie als auch von Kliniken mit der Option zur endovaskulären Therapie [24]. Eine direkte Zuweisung in ein Thrombektomiezentrum wird nach der S2e-Leitlinie und gemäß der European Stroke Organization bei Fahrzeiten um 30 min empfohlen [34]. Inwiefern ein längerer Transport in ein Thrombektomiezentrum unter Umgehung einer Stroke Unit mit der Möglichkeit einer CT-Diagnostik und Thrombolyse zu rechtfertigen ist, ohne den Patienten durch verzögerte Lyse zu gefährden, bleibt eine entscheidende Frage, die weiterhin Gegenstand wissenschaftlicher Kontroversen ist. [20, 30]. Grundlage dieser Entscheidung wird immer auch die lokale Versorgungsinfrastruktur sein. Diese sollte idealerweise gemeinsam mit einzelfallindividuellen Faktoren (patientenindividuelle Faktoren, geographische und weitere äußere Umstände von einsatztaktischer Relevanz) in validierbare Algorithmen eingebunden sein, die es dem präklinischen Team erlauben, die individuell beste Lösung für den jeweiligen Patienten im spezifischen Einsatzgeschehen zu finden. In jedem Fall sollten Transportentscheidungen im Kontext der regionalen Infrastruktur getroffen werden, unter Beachtung von Transportzeiten und tatsächlichen Prozesszeiten in den teilhabenden Kliniken [12].

Bezogen auf den direkten Transport zum Thrombektomiezentrum bleibt die Frage offen, inwiefern das Umgehen eines Krankenhauses mit CT eine Diagnosestellung der teilweise ebenfalls zeitkritischen Differenzialdiagnosen intrazerebrale Hämorrhagie oder anderer Schlaganfall-Mimics verzögern. Telemedizinischen Schlaganfallnetzwerke oder alternative Versorgungskonzepte, wie mobile Stroke Units oder mobile Intervention Teams, könnten dazu beitragen, Diagnostik und Therapie des schweren Schlaganfalls zu beschleunigen [1, 10, 13, 36].

Die Entwicklung der letzten Jahre deutet auf eine flächendeckende Ausweitung der Indikationen für die mechanische Thrombektomie hin. Diese erstreckt sich unter anderem auf distaler liegende Verschlüsse im anterioren Stromgebiet (M2- und M3-Segmente der A. cerebri media), aber auch auf Verschlüsse im posterioren Stromgebiet. Zudem weiten sich Versorgungszeitfenster zunehmend aus [21]. Zukünftige Leitlinienversionen werden diese erweiterten Indikationen mit hoher Wahrscheinlichkeit berücksichtigen, was den Rettungsdienst vor noch größere Herausforderungen zur Transportentscheidung und -logistik stellen wird. Auch aus diesem Grund sollte flächendeckend die Einführung schlaganfallspezifischer Versorgungskonzepte gefordert werden.

Nur etwa 40 % der Befragten gaben an, dass die Meldung Schlaganfall eine standardisierte Indikation für einen Notarzteinsatz darstellt, womit die Mehrzahl der Befragten formal den Vorgaben der Bundesärztekammer widerspricht. Diese hatte in ihrem Notarztindikationskatalog von 2013 den Schlaganfall explizit erwähnt [4]. Im Detail wird im Indikationskatalog jedoch auch erwähnt, dass der Notarzteinsatz vor allem bei fehlender oder deutlich beeinträchtigter Vitalfunktion erfolgen soll. Dies ist gerade bei einer milden Symptomatik (Kraftminderung einer Extremität etc.) häufig nicht gegeben. Weiterhin ist nur bei einem geringen Anteil (ca. 12 %) der Schlaganfallpatienten mit einer Verschlechterung der Symptomatik innerhalb der präklinischen Versorgungszeit zu rechnen, was dieses Vorgehen bei leichter Symptomatik unterstützt [32]. Es ist aktuell unklar, inwiefern der Einsatz ärztlichen Personals die Einordnung von Schlaganfallpatienten in Versorgungskategorien beeinflusst. Hierzu fehlen Untersuchungen, die die interprofessionelle Performance von LVO-Scores beurteilt. Dies wäre vor allem für den deutschsprachigen Raum interessant, dessen hochfrequenter Einsatz notärztlichen Personals ein internationales Alleinstellungsmerkmal darstellt.

## Fazit für die Praxis

Die vorliegende Umfrage stellt eine Bestandsaufnahme der rettungsdienstlichen Versorgungsrealität in Bezug auf die Schlaganfallversorgung in Deutschland, Österreich und der Schweiz dar. Die erheblichen Implikationen der Leitlinienanpassungen in diesem zuletzt sehr dynamischen Bereich der Notfallmedizin finden teilweise bereits Umsetzung in der Organisation der Rettungsdienstbereiche. Die Versorgungstrukturen des Rettungsdiensts können für die Betroffenen einen entscheidenden prognostischen Unterschied machen.
